# Gamma-Glutamylcyclotransferase: A Novel Target Molecule for Cancer Diagnosis and Treatment

**DOI:** 10.1155/2015/345219

**Published:** 2015-08-03

**Authors:** Susumu Kageyama, Eiki Hanada, Hiromi Ii, Keiji Tomita, Tatsuhiro Yoshiki, Akihiro Kawauchi

**Affiliations:** ^1^Department of Urology, Shiga University of Medical Science, Seta Tsukinowa-cho, Otsu, Shiga 520-2192, Japan; ^2^Department of Clinical Oncology, Kyoto Pharmaceutical University, Misasagi-Nakauchicho 5, Yamashinaku, Kyoto 607-8414, Japan

## Abstract

Gamma-glutamylcyclotransferase (GGCT) is one of the major enzymes involved in glutathione metabolism. However, its gene locus was unknown for many years. Recently, the gene for GGCT was found to be identical to C7orf24, which is registered as a hypothetical protein. Orthologs have been found in bacteria, plants, and nematodes as well as higher organisms, and the GGCT gene is highly preserved among a wide range of species. GGCT (C7orf24) was also reported as an upregulated protein in various cancers. Although the function of GGCT in cancer cells has not been determined, the following important activities have been reported: (1) high expression in various cancer tissues and cancer cell lines, (2) low expression in normal tissues, (3) inhibition of cancer cell proliferation via anti-GGCT RNAi, (4) inhibition of cancer cell invasion and migration via anti-GGCT RNAi, (5) an epigenetic transcriptional regulation in cancer cells, and (6) an antitumor effect in cancer-bearing xenograft mice. Therefore, GGCT is promising as a diagnostic marker and a therapeutic target for various cancers. This review summarizes these interesting findings.

## 1. Introduction

The existence of human gamma-glutamylcyclotransferase (GGCT) has been known since around 1970. However, its amino acid sequence and gene locus were unknown for many years [1–3]. Recently, Oakley et al. cloned cDNA encoding human GGCT and they found that GGCT is identical to the hypothetical protein C7orf24 (chromosome 7 open reading frame 24), which was previously registered as a putative open reading frame on the short arm of chromosome 7 (7p15-14) [4]. Although its function in cancer cells was unknown, several studies on C7orf24 had been previously reported by others [5–7].

Masuda et al. reported that C7orf24 is identical to cytochrome c-releasing factor (CRF21), which is a substance released into the cytoplasm when human leukemia cells U937 are treated with geranylgeraniol, an apoptosis inducer [5]. They presumed that CRF21 plays a critical role in apoptosis signaling because induction of cytochrome c release from mitochondria triggered apoptosis in HeLa cells overexpressing CRF21. Xu et al. identified 46 common cancer signature genes from a pooled DNA array database of previously reported human cancers and they reported that one of the highly expressed genes was C7orf24 [6]. We conducted proteome analysis to explore specific proteins that can be used as diagnostic markers for urothelial carcinoma, and C7orf24 was identified as a highly expressed protein in cancer tissues [7].

As mentioned above, a number of relationships between cancer and GGCT have been reported by some researchers and the role of this molecule in cancer has attracted attention. This minireview summarizes the role of GGCT in cancer cells and the possibility of using GGCT in cancer diagnosis and targeting treatment.

## 2. GGCT and Gamma-Glutamyl Cycle

**Figure 1:**
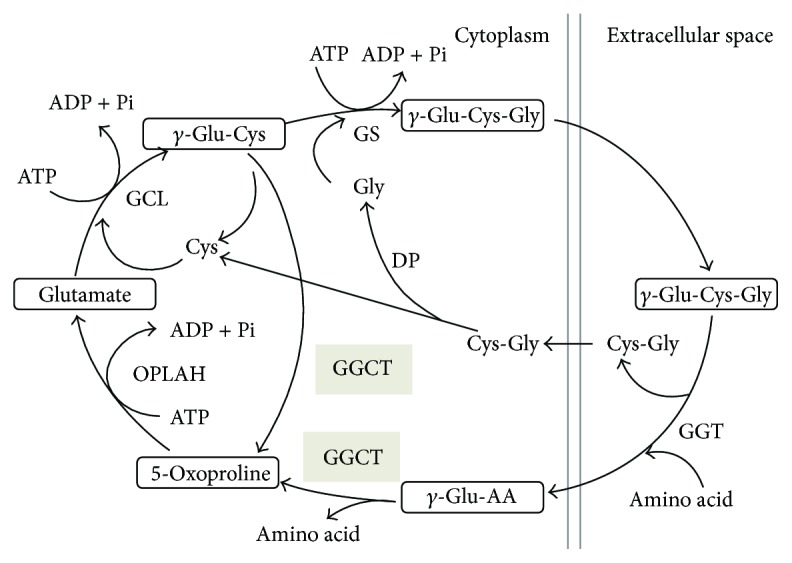
*γ*-glutamyl cycle. GGCT, gamma-glutamylcyclotransferase; GGT, gamma-glutamyltranspeptidase; GCL, glutamate cysteine ligase; GS, glutathione synthase; OPLAH, 5-oxoprolinase; *γ*-Glu-Cys-Gly, glutathione; DP, dipeptidase.

GGCT is one of the major enzymes comprising the gamma-glutamyl cycle proposed by Orlowski et al. (Figure 1) [2]. GGCT catalyzes the reaction producing 5-oxoproline and free amino acids from gamma-glutamyl peptide taken into the cell. Orthologs of GGCT range from bacteria, plants, and nematodes to higher organisms, and the GGCT gene is highly preserved among a wide range of species.

Glutathione is a tripeptide consisting of glutamate, cysteine, and glycine. Glutathione is found in cells at a relatively high concentration of 0.5–10 mM and has a number of important functions such as an antioxidant and a detoxifying agent. Adequate amounts of glutamate, cysteine, and glycine are essential to maintain glutathione at proper levels [8]. Meister et al. proposed that the gamma-glutamyl cycle plays a role in active transport of several amino acids including glutamate, cysteine, and glycine [9, 10].

Although GGCT was isolated more than 40 years ago [1–3], its gene locus was unknown until identified by Oakley et al. in 2008 [4]. Nowadays, it has gradually been learned that GGCT is expressed at a high level in a number of cancers. However, the role that GGCT, as an enzyme in the gamma-glutamyl cycle, has in the activity of cancer cells is still not known. In addition to its enzymatic role, GGCT may be a multifunctional molecule that is involved in the growth of cancer. These issues need to be clarified in future studies.

## 3. Distribution and Intracellular Localization of GGCT in Normal Tissues

Several studies concerning the distribution of GGCT in normal tissues were reported. Oda et al. examined the mRNA expression of GGCT in rats [11]. They reported that relatively high levels of mRNA were expressed in the liver and kidney but were generally lower in other organs. Oakley et al. summarized the expression profiles derived from GGCT expressed sequence tags (ESTs) using a human EST database [4]. GGCT is widely expressed in many organs and the levels of expression were higher in bladder and salivary gland than in other organs. However, the reason for this remains unknown. Regarding GGCT protein expression, Gromov et al. performed an immunohistochemical analysis using tissue microarrays [12]. They examined more than 30 normal organ samples and reported weak to moderate immunoreactivity in a wide range of normal tissues. Amano et al. also studied the immunohistochemical expression of GGCT in many normal tissues [13]. They also reported that GGCT protein was detected in most normal human tissues and mainly in epithelial cells.

The intracellular distribution found by immunohistochemistry was in the cytoplasm and nucleus and these findings were almost consistent with a few other reports [12–14]. Cytoplasmic staining by immunohistochemistry is relatively homogeneous. However, nuclear reactivity is relatively heterogeneous. Azumi et al. analyzed the cellular localization of GGCT using full-length protein and its truncated mutants [14]. They produced the transfectants (NIH3T3) that express a GFP-GGCT fusion protein or mutants. They found that the region consisting of 61–120 amino acids is required for the full-length GGCT to anchor in the cytoplasm and they elucidated that deletion of this region allows GGCT to move to the nucleus. Although the role of GGCT in the nucleus has not been determined, the GGCT observed in the nucleus may represent a posttranslationally modified form or a splice variant [13].

## 4. High Expression of GGCT in Various Cancers

We conducted proteome analysis with two-dimensional electrophoresis in bladder cancer to search for diagnostic markers for urothelial carcinomas [15, 16]. Fifteen highly expressed proteins, including GGCT, were identified through this analysis. The expression of GGCT in surgical specimens was examined by Western blot using our original monoclonal antibody, and high expression was observed in 64% and 10% of cancer and noncancerous tissues, respectively [7]. We also reported a high expression of GGCT in various human cancer cell lines other than urothelial carcinoma.

After our report, other groups reported similar results using clinical samples [12, 17, 18]. Gromov et al. conducted a large-scale proteome analysis in 123 cases of breast cancer, and they also found that GGCT was highly expressed in neoplastic as compared to normal tissues [12]. They studied the association of the expression of GGCT with the patient's outcome, and they showed that patients with high-level GGCT expression had a poor prognosis. In addition, they also studied the expression of GGCT in cancers other than breast cancer and reported that high expression of GGCT was observed in 58% of uterine cervical cancers, 38% of lung cancers, and 72% of colon cancers. Furthermore, they demonstrated that GGCT could be detected in the extracellular fluid of mammary glands and suggested the possibility of GGCT as a serum marker for breast cancer. Uejima et al. examined the expression of GGCT mRNA using 40 surgical specimens of osteosarcoma compared with normal human osteoblasts as a control. They reported a high expression (average 8.7 times higher than normal human osteoblasts) in all specimens [17]. Takemura et al. conducted an immunohistochemical examination with GGCT antibody in 200 specimens of esophageal lesions [18]. Increase of GGCT expression was observed in 87.5% of esophageal squamous cell carcinoma cases and 85.0% of high-grade intraepithelial neoplasia cases but remained at 17.5% in cases of low-grade intraepithelial neoplasia.

On the other hand, the results obtained by IHC were inconsistent in several types of cancers. Amano et al. examined 13 types of cancer specimens obtained by surgery (*n* = 30 in each cancer), and they reported significant decreased expressions were observed in renal and urothelial tumors [13]. They also reported that increased GGCT expression was not as high as in breast cancer. The reason for this discrepancy among different studies is not known. In these studies different anti-GGCT antibodies were used so their reactivity or sensitivity may not be uniform [12]. Further studies on this issue are needed.

Although some discrepant findings were observed, a high-level expression of GGCT protein was observed in a wide range of cancers. Accordingly, it is considered that GGCT has the potential to become a cancer biomarker.

## 5. Role of GGCT in Cancer Cells

Although the upregulation of GGCT in cancer cells has been reported, the role of GGCT in cancer cells is still unclear. Therefore, we performed gene transfection and knockdown experiments and demonstrated that cell proliferation is promoted by introducing the GGCT gene into NIH3T3 cells [7]. Furthermore, inhibition of cell-growth was observed by inhibiting GGCT expression with RNA interference in several types of cancer cell lines possessing high-level expressions of GGCT. In contrast, inhibition of cell proliferation was not observed in noncancer cells with very low levels of GGCT expression. Based on the above evidence, the function of GGCT related to cancer cell proliferation was suggested. Uejima et al. examined the activities of human osteosarcoma cells (HOS) using a Matrigel Invasion Chamber [17]. The migration and invasive capabilities as well as the proliferation of HOS cells were suppressed by the administration of small interfering RNA (siRNA). Furthermore, an increased expression of cell adhesion-related molecules, including integrin and cadherin, found by DNA microarray analysis of GGCT knockdown HOS cells was reported. Such results indicate the potential involvement of GGCT in not only the growth but also the invasion and metastasis of cancer.

On the other hand, we conducted a soft agar assay and focus-forming assay by introducing the GGCT gene into normal mouse fibroblasts. However, no significant changes were observed in comparison with controls [7]. Independently of our work, Azumi et al. also reported findings similar to our results by introducing the GGCT gene into HBL-100 cells, a breast cancer cell line with low-level GGCT expression [14]. Thus, it is assumed that the involvement of GGCT in malignant transformation was negated.

## 6. Molecular Regulation Mechanism of GGCT Expression in Cancer Cells

The mechanism of regulation of the GGCT gene was clarified in recent studies [19, 20]. Ohno et al. demonstrated that a region located at −371 to +14 bp of the 5′ end of GGCT is important for activation of GGCT transcription as a promoter in both cancer and noncancer cells. Sequencing analysis indicated that this region has a distinctive structure with three CCAAT boxes near the transcription start site. Moreover, a GC box exists upstream of these CCAAT boxes. NF-Y and Sp1 bind to the CCAAT boxes and GC box, respectively, to regulate positive transcription of the GGCT gene. Promoters having several NF-Y-binding CCAAT boxes were found in some genes related to the cell cycle. Accordingly, it was suggested that GGCT may also play a role in the cell cycle [19].

In addition, Ohno et al. reported a difference in GGCT gene expression between normal and cancer cells. They determined that the promoter of the GGCT gene has a stable heterochromatin structure in normal cells. In cancer cells on the other hand, the promoter has a euchromatin structure. Their report strongly suggested that high expression of GGCT in cancer cells is caused by a structural change in the chromatin of the GGCT gene associated with the oncogenic transformation of the cells [20].

## 7. Development of the GGCT-Targeting Cancer Therapy

We first reported that the administration of anti-GGCT siRNA inhibited cell proliferation in some cell lines, including bladder, prostate, lung, breast, and cervical cancers [7]. Similarly, other groups reported that the growth of cancer cells was inhibited by GGCT knockdown with RNAi* in vitro*. Recently, we found the inhibitory efficacy of the combined use of docetaxel, a standard chemotherapeutic agent for prostate cancer, with anti-GGCT siRNA using prostate cancer cell lines. The combined use of docetaxel at IC_50_ (half maximal (50%) inhibitory concentration) with GGCT siRNA at a low concentration of 5 nM in each cell line resulted in an additional 25–41% inhibition of proliferation over coadministration of docetaxel and control siRNA (manuscript in submission). The combined use of a functional inhibitor of GGCT with chemotherapy may become a promising treatment.

A study of treatment targeting GGCT using an animal model was reported by Hama et al. [21]. They produced tumor-bearing mice by subcutaneous implantation of EBC-1, a lung squamous cell carcinoma cell line, and then administered anti-GGCT siRNA to the tumor using a needle-free jet injection and obtained significant tumor regression. We also attempted to examine intravenous administration of anti-GGCT siRNA conjugated with several carrier substances in tumor-bearing mice. However, no significant therapeutic effect was obtained (data not shown). Degradation of siRNA in the blood is a common problem in systemic administration and the delivery of siRNA to the tumor was very poor. The lack of an effective drug delivery system is considered to be the critical problem in cancer treatment by intravenous administration of siRNA. Recently, however, Ran et al. succeeded in systemic cancer treatment targeting GGCT in tumor-inoculated mice via a unique drug delivery system for intravenous administration of siRNA [22]. They established the PEGylated hyaluronic acid-modified liposomal delivery system and described significant antitumor effects in mice inoculated with drug-resistant MCF-7 cells (a breast cancer cell line). Surprisingly, they also reported that a systemic administration of anti-GGCT siRNA did not affect normal organs, such as kidney, heart, lung, liver, and spleen. GGCT is expected to be a target molecule for anticancer therapy, and the development of low molecular weight inhibitors is also needed.

Recently, Yoshiya et al. developed probes “LISA-4” and “LISA-101,” which produce fluorescence as the result of an enzymatic reaction [23, 24]. The establishment of these new methods to measure GGCT activity would be very valuable to screen GGCT inhibitors.

## 8. Conclusion

It has only been a few years since the existence of GGCT was shown and accumulation of research results has not been sufficient. However, GGCT is a very attractive target molecule because its high expression has been observed in a wide range of cancer types. Inhibition of GGCT expression suppresses the proliferation of cancer cells, and mild adverse events are expected from a recent study. Further investigations are needed in the future.
